# Aryl-substituted acridanes as hosts for TADF-based OLEDs

**DOI:** 10.3762/bjoc.16.88

**Published:** 2020-05-13

**Authors:** Naveen Masimukku, Dalius Gudeika, Oleksandr Bezvikonnyi, Ihor Syvorotka, Rasa Keruckiene, Dmytro Volyniuk, Juozas V Grazulevicius

**Affiliations:** 1 Department of Polymer Chemistry and Technology, Kaunas University of Technology, Radvilenu pl. 19, LT-50254, Kaunas, Lithuania; 2 Scientific Research Company “Electron-Carat”, 202 Stryska Str. 79031, Lviv, Ukraine

**Keywords:** acridan, hole mobility, host, OLED, thermally activated delayed fluorescence

## Abstract

Four aryl-substituted acridan derivatives were designed, synthesized and characterized as electroactive materials for organic light emitting diodes based on emitters exhibiting thermally activated delayed fluorescence. These compounds possessed relatively high thermal stability with glass-transition temperatures being in the range of 79–97 °C. The compounds showed oxidation bands arising from acridanyl groups in the range of 0.31–038 V. Ionization potentials of the solid films ranged from 5.39 to 5.62 eV. The developed materials were characterized by triplet energies higher than 2.5 eV. The layer of 10-ethyl-9,9-dimethyl-2,7-di(naphthalen-1-yl)-9,10-dihydroacridine demonstrated hole mobilities reaching10^−3^ cm^2^/V·s at electric fields higher then ca. 2.5 × 10^5^ V/cm. The selected compounds were used as hosts in electroluminescent devices which demonstrated maximum external quantum efficiencies up to 3.2%.

## Introduction

Organic light emitting diodes (OLEDs) are perfect candidates for multicolor displays and for next generation energy saving large area-lighting devices [1]. Nowadays, organic compounds exhibiting thermally activated delayed fluorescence (TADF) are widely used as emitters for OLEDs [2]. The great interest in TADF emitters is mainly explained by their heavy-atoms-free molecular structure and 100% theoretical limit of internal quantum efficiency (IQE) of electroluminescent (EL) devices based on the TADF phenomenon [3]. Thus, the achievable IQE of TADF-based OLEDs is as high as it is for phosphorescent organic light emitting diodes [4]. An efficient spin conversion between triplets and singlets in organic molecules requires a small energy splitting (∆*E*_ST_) between the lowest singlet and triplet excited states [5]. Various TADF derivatives have been developed with the aim to obtain highly efficient OLEDs by combining diverse donor and electron-acceptor moieties [6–7].

To successfully exploit TADF emitters in OLED structures, appropriate hosts are required [8]. Since the selection of suitable hosts is very important for achieving high OLED efficiencies, there was considerable interest in host compounds for TADF emitters in recent years [9–10]. The host compounds for TADF-based OLEDs must match a number of censorious requests. For example, high singlet and triplet energies (higher than those of the guest) are required for host compounds for qualifying host–guest energy transfer, thus implying restrict of the emissive excitons on the TADF emitters [11]. High glass-transition temperatures are also required for increasing the morphological stability of light-emitting layers and consequently for elongation of device lifetimes [11]. Proper energy levels and bipolar charge-transporting properties of host materials may endow good charge-injection properties and charge balance in the guest–host light-emitting layers of TADF OLEDs [12–14]. Therefore, the synthesis of host materials with the combination of all required properties especially of those intended for blue TADF OLEDs is a great challenge [11,15].

Up to now, most of the compounds used as hosts in TADF-based devices demonstrate a deep HOMO energy level and shallow LUMO energy level. This poses difficulties to the injection of electrons and/or holes into the light-emitting layer [16]. This weaknesses of hosts result in low power efficiencies and high turn-on voltages of TADF-based OLEDs [17]. To overcome these challenges, several molecular design strategies were proposed including the incorporation of electron-accepting and electron-donating moieties into the same molecule [18–19]. Conventional hosts such as 1,3-bis(*N*-carbazolyl)benzene (*m*CP) and bis[2-(diphenylphosphino)phenyl] ether oxide (DPEPO), are generally used as hosts in blue phosphorescent OLEDs, are also widely applied in TADF-based OLEDs so far [20–21]. Although these hosts demonstrate relatively good results, it can be presumed that the further improvement of the performance of TADF-based OLEDs is possible, if some drawbacks of widely used hosts such as unipolar charge transport or uncomplimentary energy levels are overcome [22–26]. The only hole or electron-transporting property of hosts leads to charge recombination near the interface between the charge-transporting layer and the emissive layer, thus decreasing the device’s efficiency [27].

In this work, four acridan derivatives were prepared using simple synthetic procedures and characterized as hosts for TADF-based OLEDs.

## Results and Discussion

### Synthesis and characterization

**Scheme 1:**
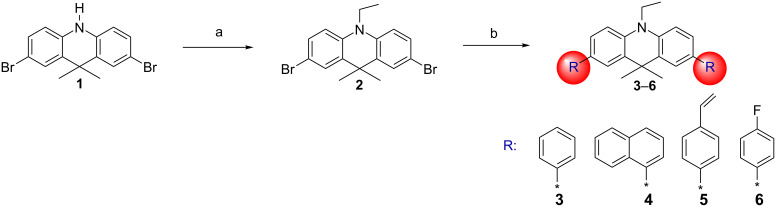
Synthesis of acridan-based compounds **3**–**6**. Reagents and conditions: (a) bromoethane, KOH, tetrabutylammonium bromide, acetone, 60 °C, 1 h; (b) napthalen-1-ylboronic acid, 4-vinylphenylboronic acid or 4-fluorophenylboronic acid, K_2_CO_3_, PdCl_2_(PPh_3_)_2_, THF/H_2_O, 80 °C, 24 h.

The synthetic routes for acridan derivatives **3–6** are outlined in Scheme 1. The key intermediate, 2,7-dibromo-9,9-dimethyl-9,10-dihydroacridine (**1**) was prepared according to the reported procedure [28]. The subsequent alkylation of **1** using bromoethane afforded the *N*-ethylated dibromo compound **2** in 80% yield. The target compounds **3–6** were then obtained by Suzuki cross-coupling reactions between brominated acridan **2** and the respective phenylboronic acids in the presence of a palladium catalyst, with yields ranging from 27 to 50%. The chemical structures of **3–6** were confirmed by ^1^H and ^13^C NMR spectroscopy, elemental analysis and mass spectrometry. Transparent thin films of these compounds could be prepared by vacuum evaporation or by spin coating from solutions.

### Theoretical calculations

**Figure 1:**
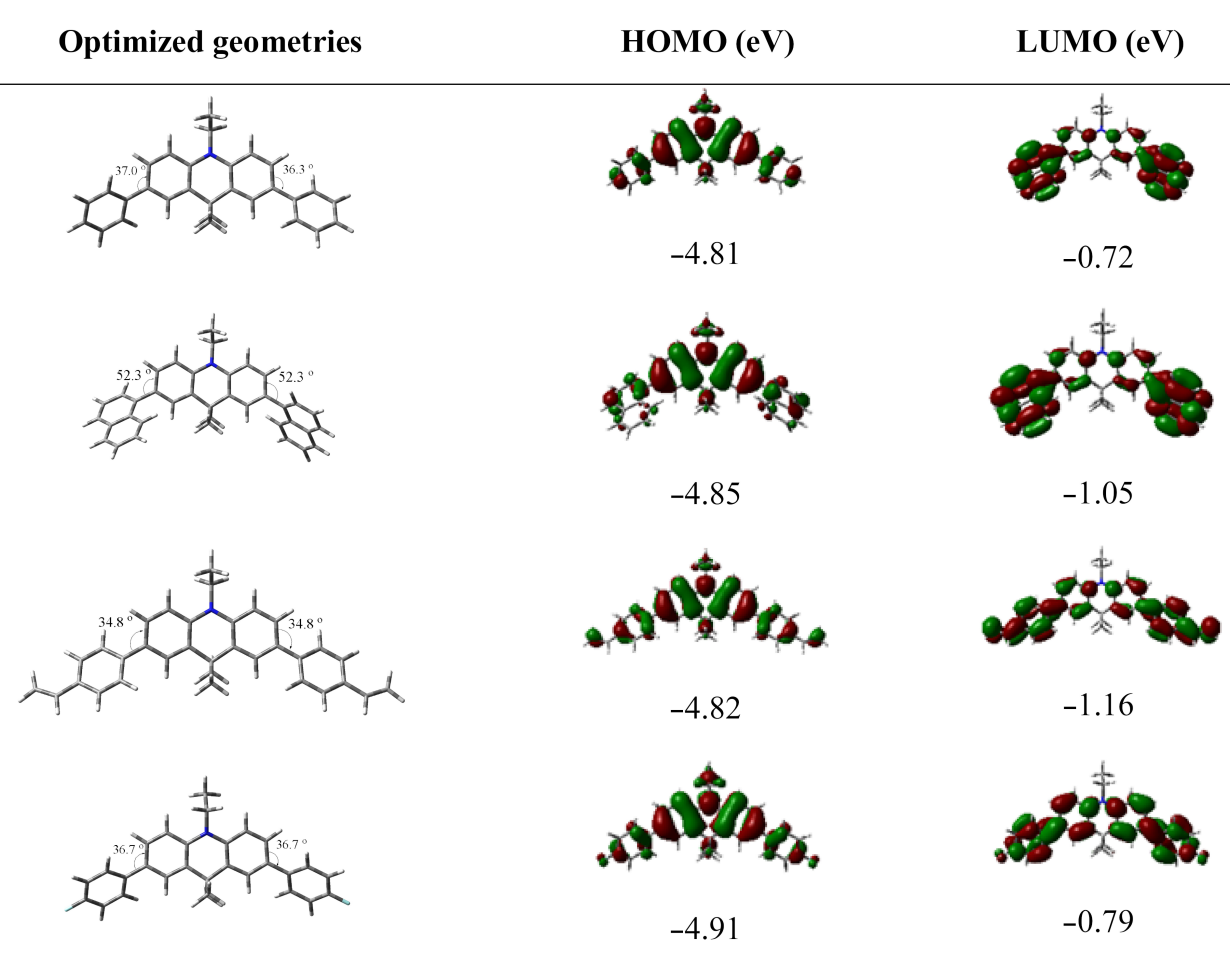
Theoretically calculated HOMO and LUMO levels distributions and optimized geometries of **3–6** DFT calculations were performed at the B3LYP/6-31G(d,p) level [29].

The optimized structures of **3–6** were obtained by density functional theory (DFT) calculations at the B3LYP/6-31G(d,p) level of theory (Figure 1). The dihedral angles between the acridanyl and phenyl moieties in compound **3** (37.0 and 36.3°) are comparable with the dihedral angles between the acridanyl and vinylphenyl or 4-fluorophenyl moieties in compounds **5** and **6** (34.8 and 36.7°, respectively). Thus, the nature of the phenyl moiety attached to the central acridan unit does not affect the dihedral angle significantly. The naphthyl-substituted acridan **4** is characterized by slightly higher dihedral angles of 52.3° as compared to compounds **3**, **5**, and **6**. The highest occupied molecular orbitals (HOMOs) and the lowest unoccupied molecular orbitals (LUMOs) of **3–6** are distributed over the entire molecules.

### Thermal properties

The behavior under heating of derivatives **3–6** was examined by DSC and TGA under a nitrogen atmosphere. The 5% mass-loss temperatures were observed in the range of 271–395 °C (Table 1 and Figure S1 in Supporting Information File 1). Compounds **3** and **6** underwent sublimation during the TGA experiments and exhibited complete weigh losses. It was therefore impossible to compare their thermal stabilities with those of compounds **4** and **5**. The latter derivatives exhibited relative high thermal stabilities with 5% mass loss temperatures of 344 and 395 °C, respectively.

**Table 1:** Thermal characteristics of acridan-based compounds **3–6**.

Compound	*T*_m_, °C (scan rate 10 °C/min)	*T*_g_, °C	*T*_cr_, °C	*T*_ID−5%_,°C

**3**	174, 174^a^	79^a^	102^a^	285
**4**	201, 201^a^	86^a^	183^a^	344
**5**	180	97^a^	242	395
**6**	186, 186^a^	–	118	271

^a^2nd heating. *T*_g_ – glass transition temperature, *T*_m_ – melting temperature, *T*_cr_ – crystallization temperature, T_ID−5%_ – 5% weight loss temperature (20 °C/min).

**Figure 2:**
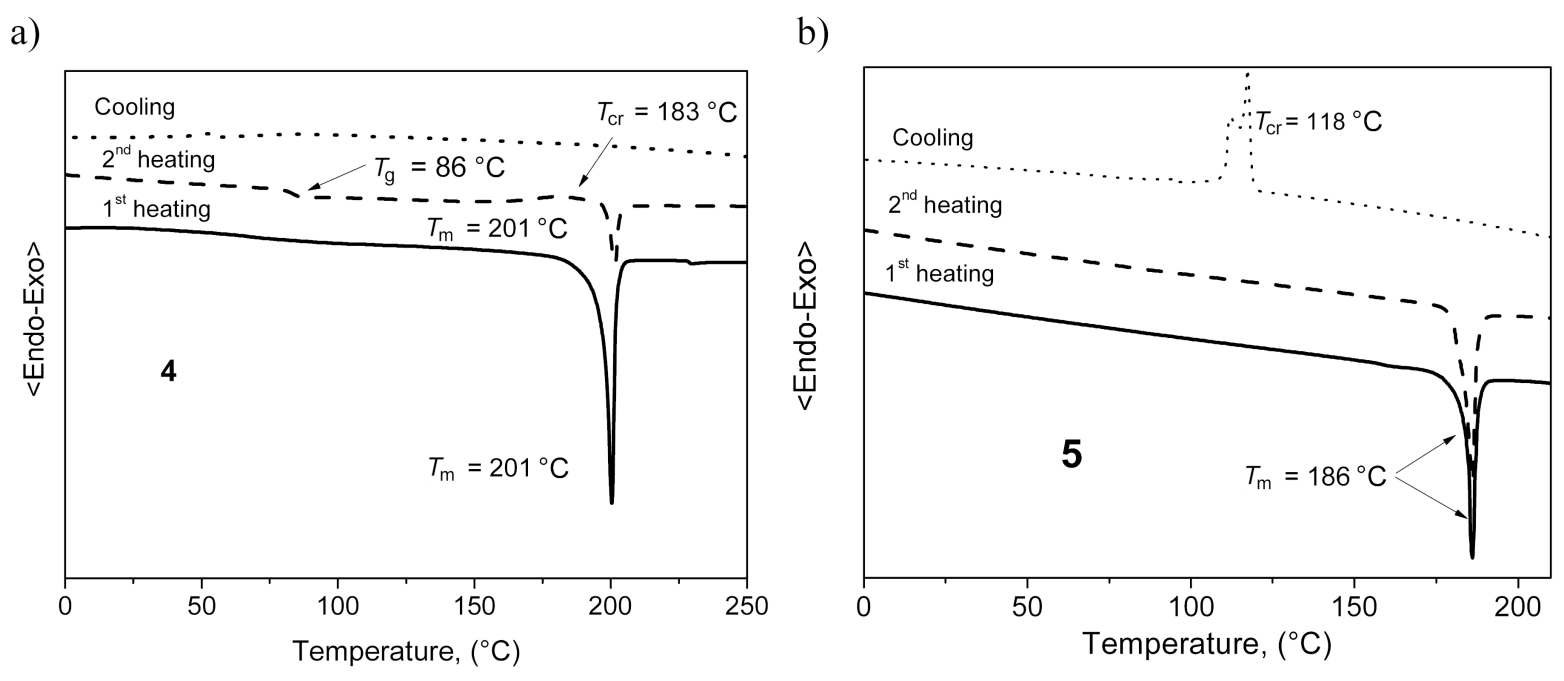
DSC curves of compounds **4** and **5**.

Compounds **3–6** were obtained as crystalline substances after the synthesis and purification. However, derivatives **3–5** could also form molecular glasses. The DSC thermograms of compound **4** are shown in Figure 2a. The crystalline sample of **4** melted at 201 °C on the first heating. The melt transformed into a solid amorphous material upon cooling. When the amorphous sample was heated on the second scan, the glass transition (*T*_g_) was noticed at 86 °C, followed by an exothermic crystallization (*T*_cr_) signal observed at 183 °C to obtain crystals, which melted at 201 °C. The crystalline sample of derivative **3** demonstrated a similar behavior. It melted upon the first heating at 174 °C and exhibited a glass transition at *T*_g_ of 79 °C in the second heating, followed by an exothermic *T*_cr_ at 102 °C. Derivative **6** demonstrated different behavior in the DSC experiments. The DSC thermograms of **6** are shown in Figure 2b. The crystalline sample of **6** melted at 186 °C on the first heating and on cooling, the melted sample crystallized at 118 °C. When the sample was heated again, only a melting peak was observed at 186 °C. The *T*_g_ values observed for compounds **3**–**5** ranged from 79 to 97 °C, with the phenyl-substituted acridan **3** exhibiting the lowest glass-transition temperature. The higher *T*_g_ values can be explained by the higher molecular weights resulting in a stronger intermolecular interaction and by larger volumes restricting molecular motion. These observations confirm that compounds **3–5** can be used for the preparation of thin amorphous layers on substrates.

### Photophysical properties

**Figure 3:**
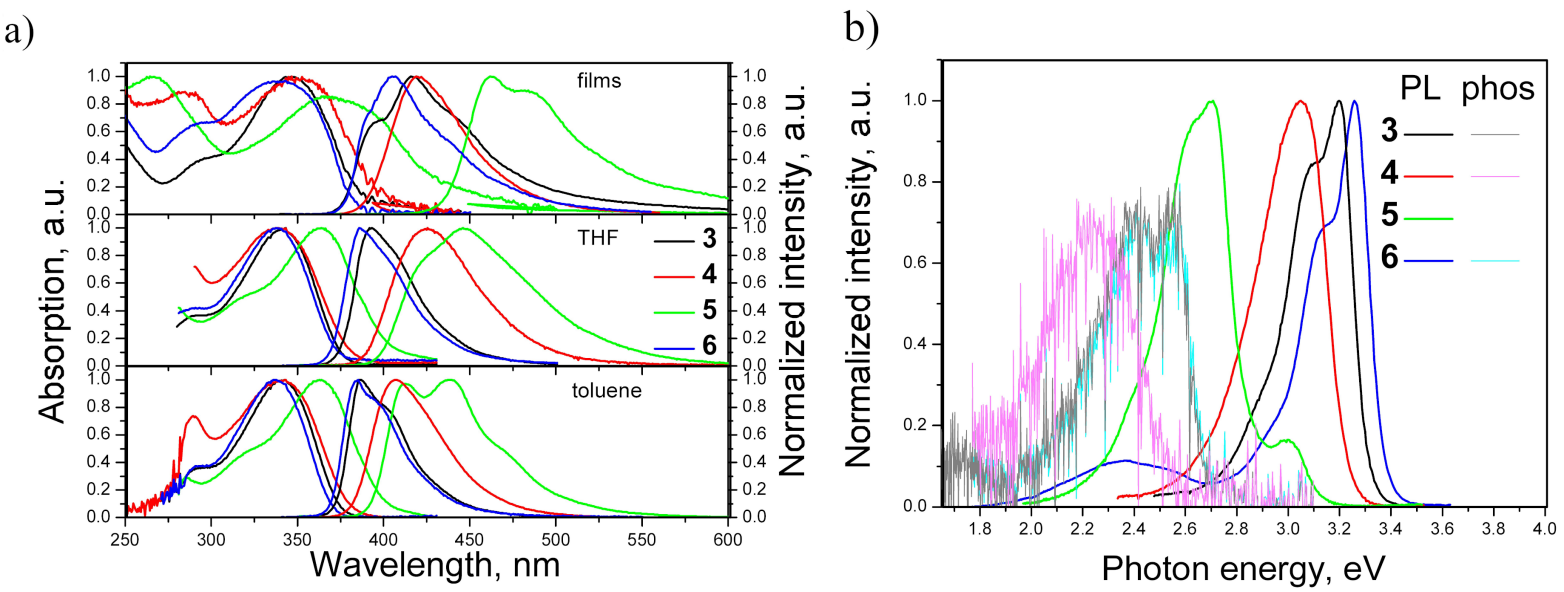
Absorption and PL spectra (λ_ex_ = 330 nm) of compounds **3**–**6**. a) Absorption spectra as neat films, dilute THF and toluene solutions. b) PL and phosphorescence spectra in dilute THF solutions at 77 K.

The absorption and photoluminescence (PL) spectra of neat films, and dilute THF and toluene solutions of the studied derivatives are presented in Figure 3a. The derivatives **3**, **4,** and **6** exhibited intense acridan-related lowest energy bands (LEB) of absorption at ≈340 nm, affected by the type of substituents. The LEB of the solutions and films of **5** were decreased by ca. 0.23 eV with respect to that of the other studied compounds due to the π-electronic coupling between the acridan and vinylphenyl moieties. The absorption spectra were not affected by the polarity of the solvent.

Relatively structured PL spectra were obtained for solutions and solid films of **3**–**6** suggesting their fluorescence resulted from emissive recombination of local excited states in nature (Figure 3a). Slight red-shifts of PL spectra were observed in higher-polar THF solutions and films of the derivatives **3**–**6** in comparison to PL spectra measured in low-polar toluene due to polarity and aggregation effects. Despite these observations, emission of compounds **3**–**6** can mainly be assigned to π–π* transitions of local excited states. However, a slight contribution of charge transfer (CT) can be also recognized in the emission of the studied compounds **3–6** (Figure 3a). The observed spectral behavior is typical for the twisted intramolecular CT phenomenon [30]. Despite the fact that the HOMOs and LUMOs are distributed over the entire molecule for all compounds, the strong electron-donating nature of acridan is manifested by a partial spatial separation of the frontier orbitals. As it can be seen from Figure 1, the LUMOs are mostly located on phenyl, naphthalenyl, vinylphenyl, and fluorophenyl moieties of the molecules. These moieties accept electrons causing a change of the dipole moments in the excited states. As a result, separate radiative processes can be accompanied by intermolecular CT state relaxation. Such emission is evidenced by solvatochromic effects, i.e., a bathochromic shift of the emission peak due to the change of the environment to a more polar one. By replacement of the solvent toluene with THF, the solution of **4** exhibited a bathochromic shift of the PL peak from 407 to 425 nm. Thus, compound **4** clearly exhibited an intermolecular CT emission. In contrast, the PL peaks related to π–π* states of the solutions of the other studied compounds were only slightly affected by the solvent replacement. The different behavior of compound **4** may be explained by the dihedral angle between the acridanyl and naphthyl moieties, that is the largest one among all the studied compounds, leading to a reduction of π-conjugation. This observation explains the distinct ICT character of the luminescence of compound **4**. The dihedral angles in the molecules of **3** and **6** are relatively small, and their LE emissions are mainly ultraviolet. There was practically no positive solvatochromism observed for the dilute solutions of compounds **3**, **5**, and **6**. Only tails related to CT can be observed in the PL spectra of these compounds. Quenching of internal molecular motion stabilizes the twist conformers stimulating the formation of intermolecular CT states. Consequently, in the PL spectra of neat films of the derivatives the emission band assigned to CT is more prominent. The PL quantum yields of neat films of compound **3**, **4**, **5**, and **6** were found to be 0.03, 0.08, 0.32 and 0.08, respectively. The relatively high PL efficiency of the film of **4** originates from the more efficient CT contribution to the emission.

The PL and phosphorescence spectra of dilute THF solutions of the studied derivatives recorded at 77 K are shown in Figure 3b. The PL spectra recorded at liquid nitrogen temperature were found to be highly similar to those recorded at room temperature (Figure 3a). The energy values of singlet (*E*_S1_) and triplet (*E*_T1_) excited states were estimated from the onsets of the spectra. The *E*_S1_ were found to be 3.31, 3.24, 3.12 and 3.37 eV for compounds **3–6**, respectively. The *E*_T1_ values were estimated as 2.54 eV for **4**, and 2.67 eV for **3** and **6**. The triplet energy could not be estimated for derivative **5**, since the phosphorescence at 77 K was practically undetectable for this compound.

### Electrochemical and photoelectrical properties

**Figure 4:**
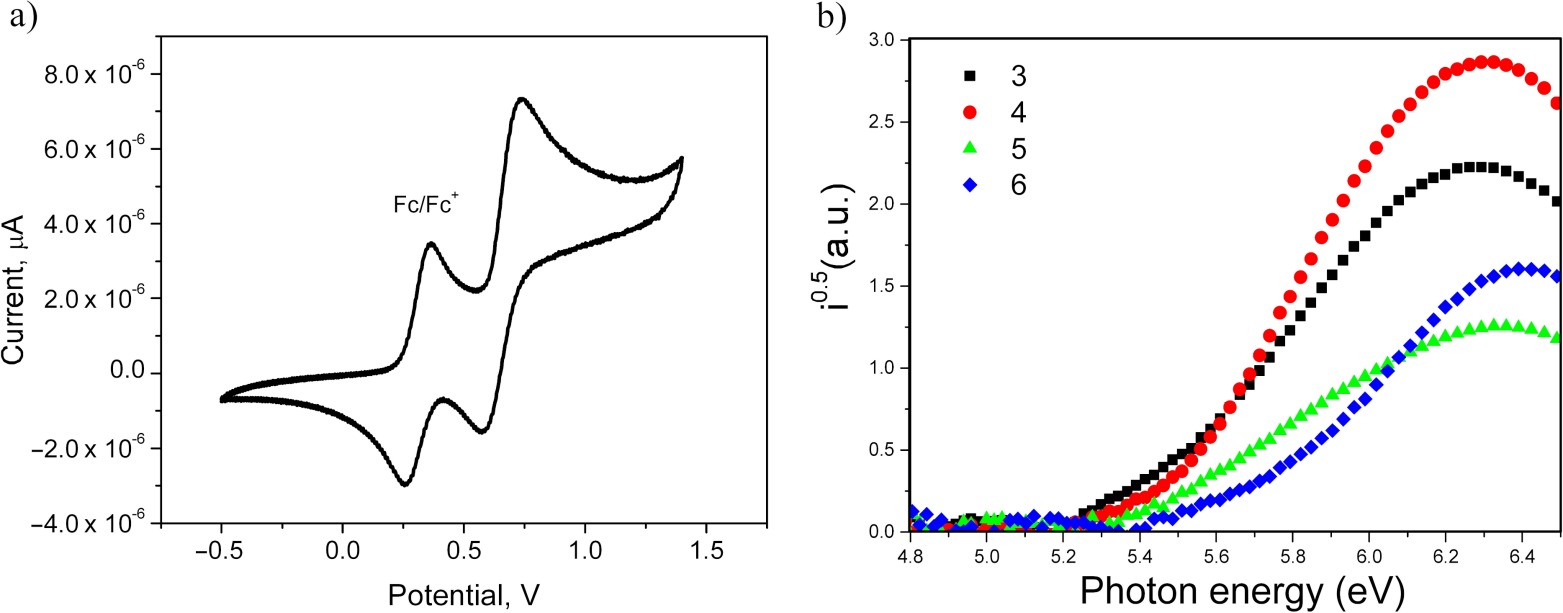
a) Cyclic voltammogram of derivative **3** in dichloromethane (a three-electrode cell consisting of a platinum coil as counter electrode, a glassy carbon working electrode, and a silver wire as reference electrode was used; sweep rate – 100 mV/s, 0.1 M solution of tetrabutylammonium hexafluorophosphate (*n*-Bu_4_NPF_6_)) and b) photoelectron emission spectra of the layers of derivatives **3**–**6**.

The electrochemical properties of the acridane derivatives **3–6** were investigated by cyclic voltammetry (CV). The cyclic voltammograms of compounds **3–6** are shown in Figure 4a and Figure S2 of Supporting Information File 1, and the data are collected in Table 2. Close values for the potentials of reversible oxidation of ca. 0.3–0.4 V were observed for the compounds. During the anodic oxidation sweeps, compounds **3–6** showed single reversible oxidation speaks, which could be tributed to the oxidation of the acridanyl moiety. Also close values of ionization potentials (IP_cv_) of the compounds **3–6** obtained from the onset potentials of their oxidation signals were found in the range of 5.11–5.18 eV. The electron affinity values were deduced from the IP_cv_ and energy gap (*E*_g_^opt^) obtained from the onsets of the UV–vis absorption spectra.

**Table 2:** Electrochemical properties of compound **3–6**.

Compound	*E*_ox_^a^, V	IP_CV_^b^, eV	*E*_g_^opt c^, eV	EA_CV_^d^, eV	IP_PES_^e^, eV	*E*_g_^opt f^, eV	EA_PES_^g^, eV

**3**	0.35	5.15	3.29	1.86	5.4	3.19	2.21
**4**	0.36	5.16	3.25	1.91	5.46	3.1	2.36
**5**	0.31	5.11	3.25	1.86	5.39	2.8	2.59
**6**	0.38	5.18	3.41	1.77	5.62	3.25	2.37

^a^Onset oxidation potential versus Ag/Ag^+^; ^b^calculated using formula IP_CV_ = (*E*_ox_ − *E*_Fc/Fc_^+^) + 4.8 (eV); ^c^estimated from an onset wavelength (λ_edge_) of absorption spectra for toluene solutions using an empirical formula *E*_g_^opt^ = 1240/_λedge_; ^d^calculated using the formula EA_CV_ = IP_CV_ − *E*_g_^opt^; ^e^obtained from PES spectra; ^f^estimated for solid films (Figure 3a); ^g^calculated using the formula EA_PES_ = IP_PES_ − *E*_g_^opt^.

The ionization potentials IP_PES_ of the solid films of derivatives **3**–**6** were estimated by photoelectron emission spectrometry (PES, Figure 4b, and Table 2). The IP_PES_ values were further used for constructing OLED structures. The highest IP_PES_ of 5.62 eV was obtained for compound **6** which contains electron-accepting fluorine atoms. The other compounds (**3–5**) demonstrated similar IP_PES_ values mainly attributed to removing an electron from the acridan unit. Slightly higher IP_PES_ values were obtained by PES measurements for compounds **3–6** in comparison to those estimated by CV and can apparently be explained by a more difficult removal of electrons from materials in the solid state than in solution due to the strong intermolecular interaction.

### Charge transporting properties

**Figure 5:**
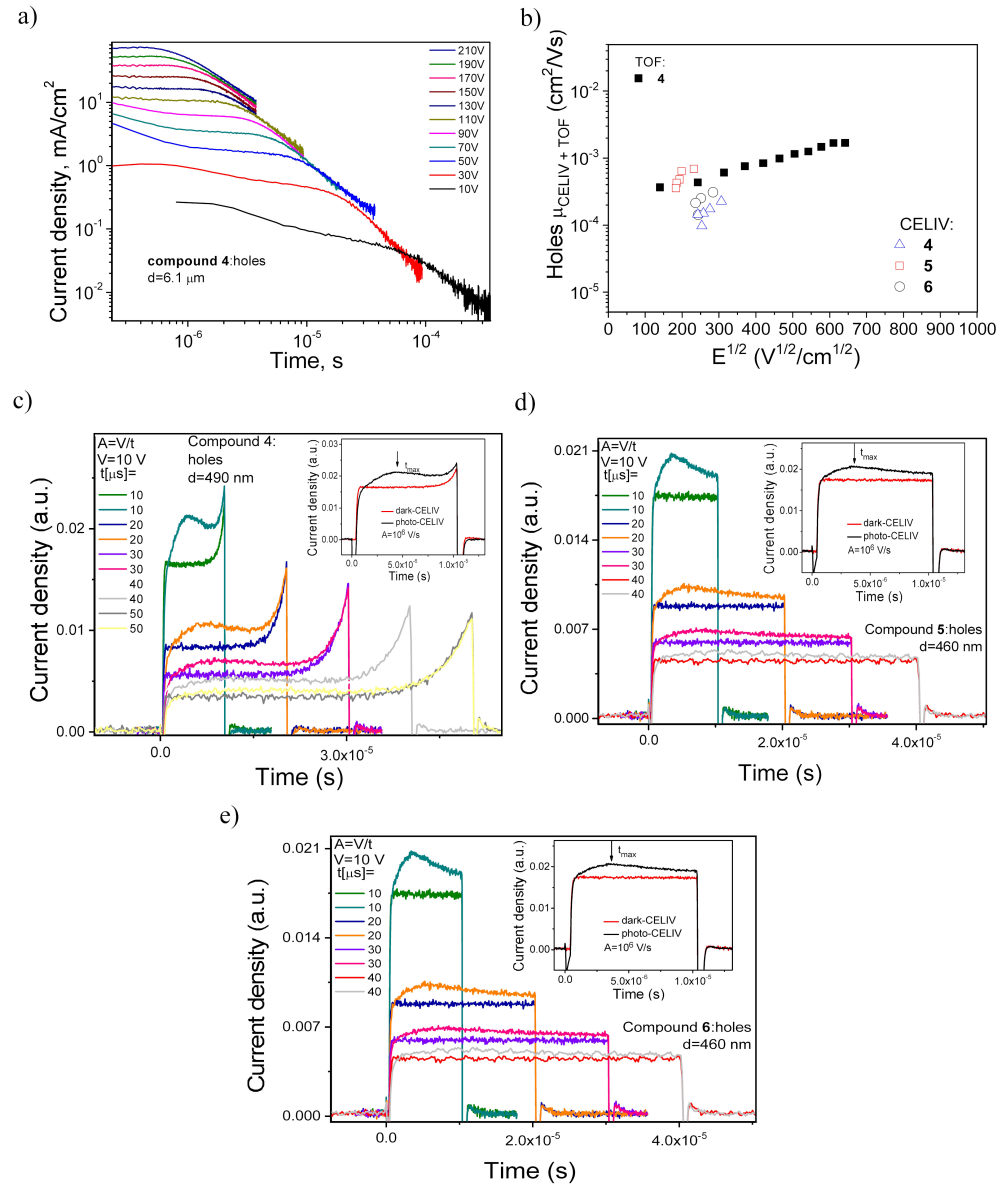
TOF photocurrent transients for holes in vacuum-deposited layers of compound **4** (a); hole mobility versus electric field for layers of compounds **4–6** (b); dark-CELIV and photo-CELIV signals for compounds **4** (c), **5** (d), and **6** (e).

To unclose the potential of acridan-based derivatives containing phenyl or naphthyl substituents as hosts for blue TADF OLEDs, charge-transport properties of the vacuum deposited layers of the acridanes were tested by the methods of time-of-flight (TOF) and charge extraction by linearly increasing voltage (CELIV) [31–32]. TOF photocurrent transients with well-visible transit times were recorded for holes in layers of compound **4** (Figure 5a). Using the values of transit times, hole-drift mobilities at different electric fields were calculated and plotted in Figure 5 according to the Poole–Frenkel model µ = µ_0_ exp(β·*E*^0.5^)*,* where µ and µ_0_ are respectively hole and field-free mobilities, β is the Poole–Frenkel constant, and *E* is the electric field [31]. The values of hole mobility in the layers of **4** exceeded 10^−3^ cm^2^/V·s at electric fields higher than ca. 2.5 × 10^5^ V/cm. Electron transport was not detected for the tested samples by TOF experiments. TOF photocurrent transients with well-visible transit times for the samples **3**, **5**, and **6** were not observed apparently due to either strong crystallinity of thick films or strongly dispersive transport of charges. Therefore, the CELIV method, which is less sensitive to charge-transport dispersity than the TOF method, was additionally exploited for charge-transport characterization of the compounds. Indeed, well discernible maxima were observed not only for the layers of compound **4** but also for the layers of **5** and **6** (Figure 5c,d and Figure S3 in Supporting Information File 1). Close values of hole mobilities in the layers of compound **4** were obtained by both the methods (Figure 5b). The similar hole mobilities were also obtained for compounds **5** and **6** by the CELIV measurements (Figure 5b). Thus, a negligible effect of the nature of substituents of acridan in compounds **4–6** on the hole-transporting properties was detected.

### OLED fabrication and characterization

**Figure 6:**
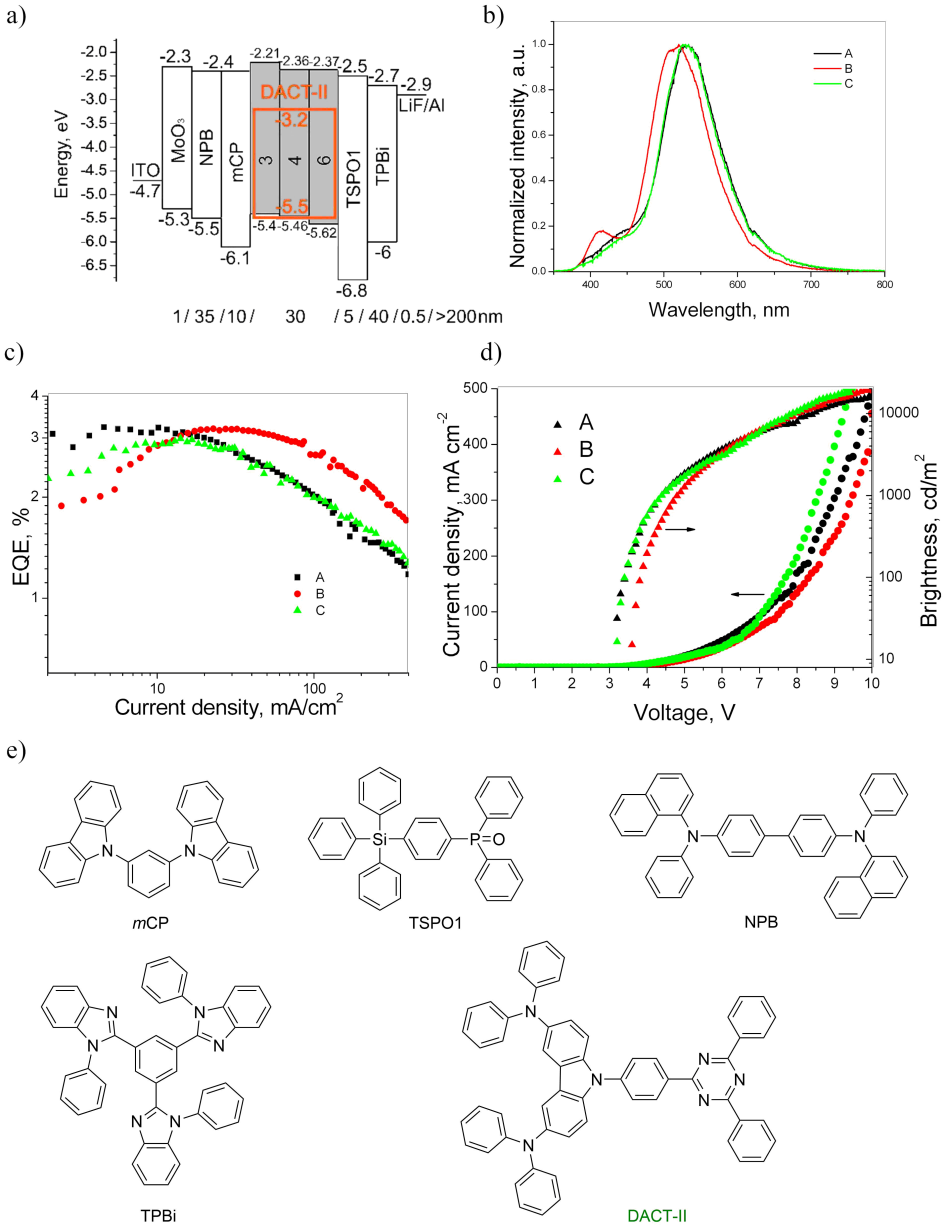
Energy diagrams of the fabricated OLEDs (a); normalized electroluminescence spectra of devices **A–C** recorded at 5 V (b); EQE versus current density plots (c), brightness and current density versus applied voltages plots (d) of the tested OLEDs; molecular structures of the organic derivatives used in the devices (e).

To test the studied compounds as host compounds in OLEDs, devices based on the well-known TADF emitter 9-[4-(4,6-diphenyl-1,3,5-triazin-2-yl)phenyl]-*N*^3^*,N*^3^*,N*^6^*,N*^6^-tetraphenyl-9*H*-carbazole-3,6-diamine (DACT-II) were fabricated and characterized [33]. The DACT-II-based OLEDs are expected to reach an internal quantum efficiency (IQE) of 100%. The low-energy absorption bands at ≈410 nm of the film of the emitter DACT-II and the emission bands of the films of the studied compounds overlapped to a greater extent in case of derivatives **3**, **4**, and **6** than in case of compound **5**, that showed an unsuitably red-shifted emission. Taking this observation into account, DACT-II (10 wt %) was used as the emitter doped into hosts **3**, **4**, and **6** in OLEDs **A**, **B** and **C**, respectively. The structures and equilibrium energy diagrams of the devices are presented in Figure 6a. The values of ionization potentials and electron affinities of solid samples of compounds **3**, **4**, and **6** were taken as HOMO and LUMO levels as the first approximation (Figure 4b, Table 2). In the devices, MoO_3_ and LiF were employed as materials for injection layers for holes and electrons, respectively. *N*,*N*'*-*Di(1-naphthyl)-*N,N*'*-*diphenyl-(1,1'-biphenyl)-4,4'-diamine (NPB) was used for the preparation of the hole-transporting layer. 1,3-Bis(*N*-carbazolyl)benzene (*m*CP) was selected as exciton blocking material. Diphenyl-4-triphenylsilylphenylphosphine oxide (TSPO1) was used as hole blocking material, while the layer of 2,2',2"-(1,3,5-benzenetriyl)-tris(1-phenyl-1*H*-benzimidazole) (TPBi) was employed as the electron-transporting layer. Electroluminescence (EL) spectra of devices **A–C** and their external quantum efficiencies (EQE) are presented in Figure 6b,c and Figure S4 (Supporting Information File 1). The current density–voltage and luminance curves, the current and power efficiencies are shown in Figure S5 (Supporting Information File 1). The characteristics of the fabricated OLEDs are collected in Table 3.

**Table 3:** Electroluminescence characteristics of OLEDs.

Device	Turn-on voltage, V	Maximum brightness, 10^3^ cd/m^2^	External quantum efficiency (EQE) maximum, %	Maximum power efficiency (PE), lm/W	EQE and (PE) efficiencies at 100 cd/m^2^	CIE 1931 UCS coordinates at 9 V

**A**	3.2	16.2	3.2	9.5	3.1% (8.8 lm/W)	(0.29, 0.5)
**B**	3.6	22.9	3.2	5.6	1.8% (4.4 lm/W)	(0.24, 0.47)
**C**	3.2	18.6	3	7.2	2% (5.9 lm/W)	(0.28, 0.51)

The intensity maxima of the EL spectra recorded at 5 V for devices **A–C** were found in the narrow range from 520 to 530 nm due to the slight differences in the dipole moments of the hosts used (Figure 6b). Additional, low-intensity peaks in the violet/blue region of the EL spectra of devices **A** and **B** appeared illustrating an incomplete energy transfer from hosts **3**, **4**, and **6** to DACT-II in the emitting layers of devices **A–C**. However, the changes in EL colors of the fabricated devices were not significant meaning that the EL spectra represent DACT-II emission according to the corresponding CIE coordinates (Table 3).

Low turn-on voltages of 3.2–3.6 V recorded for devices **A–C** indicate good charge-injecting and charge-transporting properties of the hosts used due to their lower HOMO in comparison to that of *m*CP (turn-on voltage of 3.7 V was observed for device **M**, Figure 6d, and Table 3). Close values of maximum EQEs were obtained for devices **A–C** displaying similar host performances of the compounds **3**, **4** and **6** (Figure 6c, Table 3). Maximum EQE values of 3–3.2% were observed for devices **A–C**. The rather low EQE values of devices **A–C** can be explained by the following reasons: 1) incomplete energy transfer from hosts to the guest; 2) formation of a recombination zone near to the light-emitting layer/hole-blocking layer interface due to the unipolar hole mobility of the synthesized hosts; 3) poor balance of holes and electrons in the light-emitting layer, etc. The results obtained suggest that the developed compounds could be more appropriate for an application as hole-transporting materials in organic optoelectronic devices.

## Conclusion

Four new aryl-substituted derivatives of acridan were designed and synthesized as electroactive materials for organic light emitting diodes. The thermal, photophysical, and electrochemical properties of the compounds were investigated. Most of the compounds formed glasses with glass-transition temperatures in the range of 79–97 °C. The triplet energies of the developed compounds were higher than 2.5 eV. The ionization potentials of their solid films were found in the range from 5.39 to 5.62 eV by photoelectron emission spectroscopy. For one compound, the hole mobility exceeded 10^−3^ cm^2^/V·s at electric fields higher than 2.5 × 10^5^ V/cm. The selected compounds demonstrated similar host performances in electroluminescent devices with low turn-on voltages of 3.2–3.6 V and maximum external quantum efficiencies of 3.0–3.2%.

## Experimental

### Reagents

9,9-Dimethyl-9,10-dihydroacridine, bromoethane, tetrabutylammonium bromide, phenylboronic acid, napthalen-1-ylboronic acid, 4-vinylphenylboronic acid, and 4-fluorophenylboronic acid were purchased from Fluorochem or Aldrich and were used as received. 2,7-Dibromo-9,9-dimethyl-9,10-dihydroacridine (**1**) was synthesized according to the reported procedure [28].

### Instrumentation

Differential scanning calorimetry (DSC), thermogravimetric analysis (TGA) measurements, and acquisition of mass (MS), infrared (IR), and elemental spectra were carried out as described earlier [34]. ^1^H NMR and ^13^C NMR spectra were obtained using a Varian Unity Inova (300 MHz (^1^H) and 75 MHz (^13^C)). Absorption and photoluminescence (PL) spectra of dilute solutions and of the films were recorded as described previously [35]. Theoretical calculations were carried out using Gaussian 16 [29] and Gaussview 6 software. The ionization potential measurements of the solid samples were performed by the photoelectron emission method in air [36]. Cyclic voltammetry (CV) measurements of the liquid samples were carried out as described earlier [37]. Charge drift mobility measurements for the studied compounds were performed by two methods, i.e., time-of-flight (TOF) and charge extraction by linearly increasing voltage (CELIV) in the photo regime [38]. OLEDs were fabricated by vacuum deposition of inorganic and organic layers onto cleaned ITO-coated glass, applying vacuum of 10^−6^ Torr. The active area of the obtained devices was 3 × 6 mm^2^, furthermore measurement was made after the creation of the device, in the air without passivation. The luminance voltage and current density voltage dependencies were measured with a brightness and semiconductor parameters analyzer (HP 4145A) using a calibrated photodiode and electroluminescence spectra were recorded with an Ocean Optics modular spectrometer [39].

### Synthesis

**2,7-Dibromo-10-ethyl-9,9-dimethyl-9,10-dihydroacridine (2).** 2,7-Dibromo-9,9-dimethyl-9,10-dihydroacridine (0.7 g, 1.9 mmol) was dissolved in acetone (25 mL), tetrabutylammonium bromide (0.06 g, 0.1 mmol) and potassium hydroxide (0.31 g, 5.7 mmol) were added and the mixture stirred for 30 min. Then, bromoethane (0.31 g, 2.85 mmol) was added dropwise to the reaction mixture with constant stirring and the mixture refluxed for 1 h. The reaction mixture was then poured into ice water (250 mL) with vigorous stirring. After filtration and crystallization from methanol compound **2** was obtained as white crystals. Yield (0.60 g, 80%); mp 83–84 °C; ^1^H NMR (400 MHz, CDCl_3_) δ 7.46 (d, *J* = 2.3 Hz, 2H), 7.30 (dd, *J* = 8.7, 2.3 Hz, 2H), 6.84 (d, *J* = 8.7 Hz, 2H), 3.98 (q, *J* = 7.0 Hz, 2H), 1.48 (s, 6H), 1.38 (t, *J* = 7.0 Hz, 3H); ^13^C NMR (101 MHz, CDCl_3_) δ 206.9, 139.1, 133.8, 129.4, 127.4, 114.0, 113.1, 40.4, 36.4, 30.9, 28.6, 11.4; MS (APCI^+^, 20 V) *m*/*z*: 396 ([M + H]^+^).

**10-Ethyl-9,9-dimethyl-2,7-diphenyl-9,10-dihydroacridine (3).** 2,7-Dibromo-10-ethyl-9,9-dimethyl-9,10-dihydroacridine (0.3 g, 0.75 mmol), phenylboronic acid (0.2 g, 1.57 mmol), K_2_CO_3_ (0.3 g, 2.5 mmol), and PdCl_2_(PPh_3_)_2_ (0.021 g, 0.03 mmol) were dissolved in a mixture of THF and water under argon. The resulting solution was heated at 80 °C for 24 h. After cooling to room temperature, the solution was mixed with 150 mL of water and the product extracted with dichloromethane. The obtained crude product was purified by column chromatography using ethyl acetate/*n*-hexane 1:20 as the eluent, recrystallized from the mixture of eluent to afford the target compound **3** as white crystals (0.15 g, 50%). Mp 170–171 °C; ^1^H NMR (400 MHz, CDCl_3_) δ 7.67 (s, 2H), 7.63–7.56 (m, 4H), 7.48–7.39 (m, 6H), 7.36–7.25 (m, 2H), 7.13–7.02 (m, 2H), 4.18–4.00 (m, 2H), 1.65 (s, 2H), 1.48 (t, *J* = 7.0 Hz, 3H); ^13^C NMR (101 MHz, CDCl_3_) δ 141.3, 139.4, 128.7, 126.5, 126.4, 125.2, 123.23, 112.7, 40.7, 36.1, 29.4, 29.0, 11.4; MS (APCI^+^, 20 V) *m*/*z*: 390 ([M + H]^+^); anal. calcd for C_29_H_27_N: C, 89.42; H, 6.99; N, 3.60; found: C, 89.46; H, 7.03; N, 3.62%.

**10-Ethyl-9,9-dimethyl-2,7-di(naphthalen-1-yl)-9,10-dihydroacridine (4).** Compound **4** was obtained as white crystals following the analogous procedure as described for **3** using napthalen-1-ylboronic acid (0.28 g, 1.6 mmol) instead of phenylboronic acid. The crude product was purified by silica gel column chromatography with THF/*n*-hexane 1:20 as the eluent and recrystallized from the mixture of eluent to get the target compound **4** as white crystals (0.14 g, 41%). Mp 195–196 °C; ^1^H NMR (400 MHz, CDCl_3_) δ 8.01 (d, *J* = 8.4 Hz, 2H), 7.90 (d, *J* = 7.6 Hz, 2H), 7.84 (d, *J* = 8.3 Hz, 2H), 7.58 (d, *J* = 1.9 Hz, 2H), 7.54–7.37 (m, 10H), 7.16 (d, *J* = 8.4 Hz, 2H), 4.20 (q, *J* = 6.9 Hz, 2H), 1.62 (s, 6H), 1.56 (t, *J* = 6.9 Hz, 3H); ^13^C NMR (101 MHz, CDCl_3_) δ 140.5, 139.4, 133.9, 132.7, 131.9, 131. 7 128.3, 128.2 127.1, 126.7, 126.4 126.2, 125.9, 125.6, 124.4 112.2, 40.6, 36.4, 29.7, 29.4, 11.9; MS (APCI^+^, 20 V) *m*/*z*: 490 ([M + H]^+^); anal. calcd for C_37_H_31_N: C, 90.76; H, 6.38; N, 2.86; found: C, 90.81; H, 6.42; N, 2.91%.

**10-Ethyl-9,9-dimethyl-2,7-bis(4-vinylphenyl)-9,10-dihydroacridine (5).** Compound **5** was synthesized as white crystals following the analogous procedure as described for **3** using 4-vinylphenylboronic acid (0.28 g, 1.6 mmol) instead of phenylboronic acid. The crude product was purified by silica gel column chromatography with THF/*n*-hexane 1:4 as the eluent and recrystallized from the mixture of eluent to get the target derivative **5** as white crystals (0.09 g, 27%). Mp 177–178 °C; ^1^H NMR (400 MHz, CDCl_3_) δ 7.67 (d, *J* = 3.7 Hz, 2H), 7.57 (d, *J* = 8.2 Hz, 3H), 7.49–7.45 (m, 5H), 7.28–7.22 (m, 1H), 7.19–7.13 (m, 1H), 7.07 (d, *J* = 8.2 Hz, 2H), 6.79–6.70 (m, 2H), 5.78 (d, *J* = 10.9 Hz, 2H), 5.25 (d, *J* = 10.9 Hz, 2H), 4.11 (q, *J* = 7.0 Hz, 2H), 1.65 (s, 6H), 1.47 (t, *J* = 7.0 Hz, 3H); ^13^C NMR (101 MHz, CDCl_3_) δ 140.7, 139.4, 136.5, 135.8, 132.7, 132.3, 129.0, 128.2, 126.6, 126.5 125.2, 123.3, 113.4, 112.7, 40.4, 36.4, 29.5, 21.4, 11.7; MS (APCI^+^, 20 V) *m*/*z*: 442 ([M + H]^+^); anal. calcd for C_33_H_31_N: C, 89.75; H, 7.08; N, 3.17; found: C, 89.79; H, 7.12; N, 3.22%.

**10-Ethyl-2,7-bis(4-fluorophenyl)-9,9-dimethyl-9,10-dihydroacridine (6).** Derivative **6** was synthesized as white crystals following the analogous procedure as described for **3** using 4-fluorophenylboronic acid (0. 28 g, 1.6 mmol) instead of phenylboronic acid. The crude product was purified by silica gel column chromatography with ethyl acetate/*n*-hexane 1:10 as the eluent and recrystallized from the mixture of eluent to afford the target compound **6** as white crystals (0.1 g, 34%). Mp 180–181 °C; ^1^H NMR (400 MHz, CDCl_3_) δ 7.60 (d, *J* = 2.1 Hz, 2H), 7.55–7.51 (m, 4H), 7.40 (dd, *J* = 8.4, 2.1 Hz, 2H), 7.14–7.03 (m, 6H), 4.11 (q, *J* = 7.0 Hz, 2H), 1.63 (s, 6H), 1.47 (t, *J* = 7.0 Hz, 3H); ^13^C NMR (101 MHz, CDCl_3_) δ 160.8, 139.4, 137.2, 132.3, 132.1, 128.6, 128.5, 128.0, 127.9, 127.2, 127.0, 125.3, 123.3, 115.5, 115.4, 114.2, 113.7, 112.7, 40.4, 36.4, 29.3, 11.7; MS (APCI^+^, 20 V) *m*/*z*: 426 ([M + H]^+^); anal. calcd for C_29_H_25_F_2_N: C, 81.86; H, 5.92; F, 8.93; N, 3.29; found: C, 81.91; H, 5.99; N, 3.31%.

## Supporting Information

File 1 Charge drift mobility measurements, TGA curves of **3–6**, cyclic voltammetry data of **4–6**, TOF and CELIV current transients for **4**, **5**, and **6**, current efficiency, and power efficiency versus current density for the tested OLEDs.
