# Supplementary material for: Aryl-substituted acridanes as hosts for TADF-based OLEDs
Source: Beilstein J Org Chem. 2020 May 13;16:989–1000. doi: 10.3762/bjoc.16.88 (PMC7237810; doi:10.3762/bjoc.16.88)
Supplement: File 1 — Charge drift mobility measurements, TGA curves of 3–6, cyclic voltammetry data of 4–6, TOF and CELIV current transients for 4, 5, and 6, current efficiency, and power efficiency versus current density for the tested OLEDs. [file Beilstein_J_Org_Chem-16-989-s001.pdf]

BEILSTEIN JOURNAL OF ORGANIC CHEMISTRY

Supporting Information

for

Aryl-substituted acridanes as hosts for TADF-based OLEDs

Naveen Masimukku, Dalius Gudeika, Oleksandr Bezvikonnyi, Ihor Syvorotka, Rasa Keruckiene, Dmytro Volyniuk and Juozas V. Grazulevicius

Beilstein J. Org. Chem. **2020,** *16,* 989–1000. doi:10.3762/bjoc.16.88

Charge drift mobility measurements, TGA curves of 3–6, cyclic voltammetry data of 4–6, TOF and CELIV current transients for 4, 5, and 6, current efficiency, and power efficiency versus current density for the tested OLEDs

License and Terms: This is a supporting information file under the terms of the Creative Commons Attribution License (http://creativecommons.org/licenses/by/4.0). Please note that the reuse, redistribution and reproduction in particular requires that the authors and source are credited. The license is subject to the *Beilstein Journal of Organic Chemistry* terms and conditions: (https://www.beilstein-journals.org/bjoc)

Supporting Information

Charge drift mobility measurements

Sandwich-like structures (an indium tin oxide (ITO) anode/vacuum deposited layer based on the studied compound/Al cathode) with different thicknesses of the tested layers and the active area of 6 mm^2 were fabricated for TOF and CELIV measurements. Obtained the Lumtec from company, ITO glasses 15 Ω were patterned and cleaned before use. Then, the tested layers and Al cathode were evaporated at 5–10 Å/s at a pressure below 2×10^{-6} mbar. The thickness (d) of the layers was obtained by CELIV measurement assuming the dielectric constant of 3 for the tested materials [1]. In both, TOF and CELIV measurements, a 355 nm wavelength laser (EKSPLA NL300) excitation was utilized to generate charges on the surface of the tested layers from the ITO side. In the TOF measurement, positive and negative external voltages (U) were applied to the samples for checking hole and electron transport employing a 6517B electrometer (Keithley). Triangle pulses which were synchronised with the laser pulse in the photo CELIV measurements were applied to the samples using the delay generator AFG3011C (Tektronix). The oscilloscope TDS3032C (Tektronix) was used to record the TOF and CELIV photocurrent transients under the different electric fields for the transported charges. Charge mobilities were evaluated from the equation $\mu = d^2/(U \cdot t_{tr})$ using transit times (t_{tr}) taken from TOF photocurrent transients. The transit time t_{tr} for the samples with the charge transporting material was determined by the kink on the curve of the transient in log-log scale. Since charges were generated at the surface of the tested layers in the CELIV experiment, the carrier mobility was calculated by the followed equation $\mu = \frac{2d^2}{At_{max}^2}$. Here, the t_{max} is the time for the extraction current reaching its maximum, when characteristic maximum is observed, $A = U(t)/t$ is the voltage increase rate, U(t) and t are the amplitude and period of the triangle pulses, respectively.

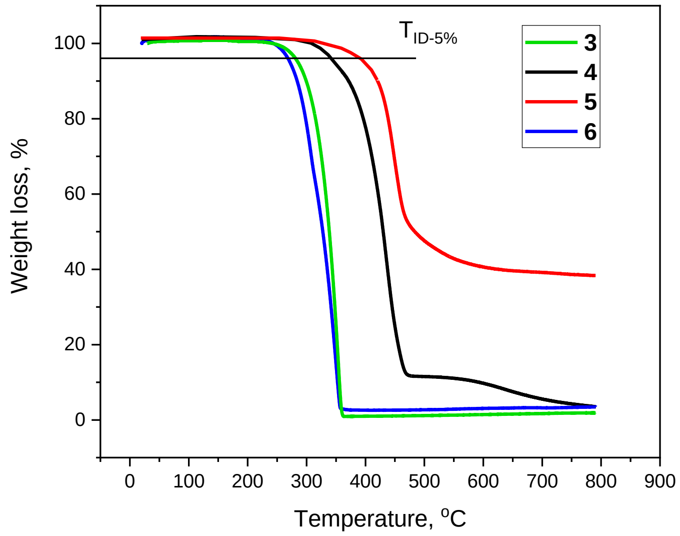

Figure S1: TGA curves of compounds **3–6**.

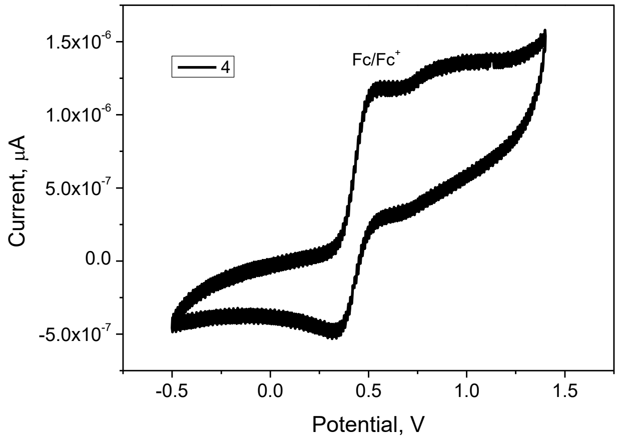

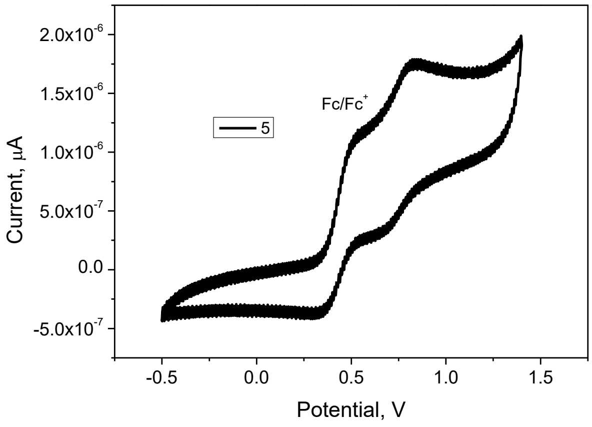

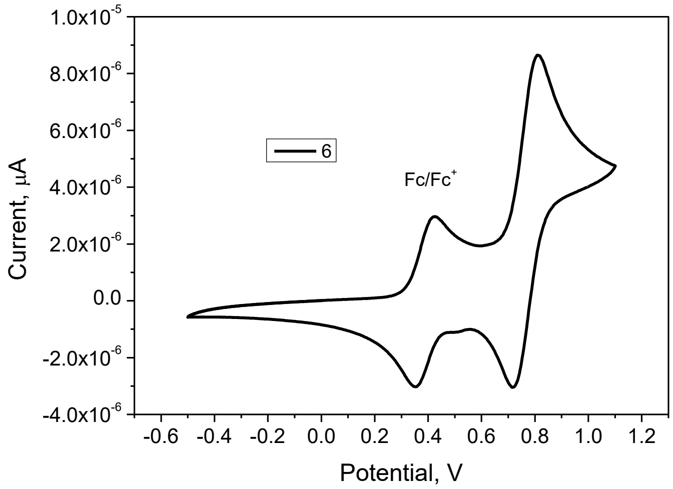

Figure S2: Cyclic voltammograms of compounds **4–6**.

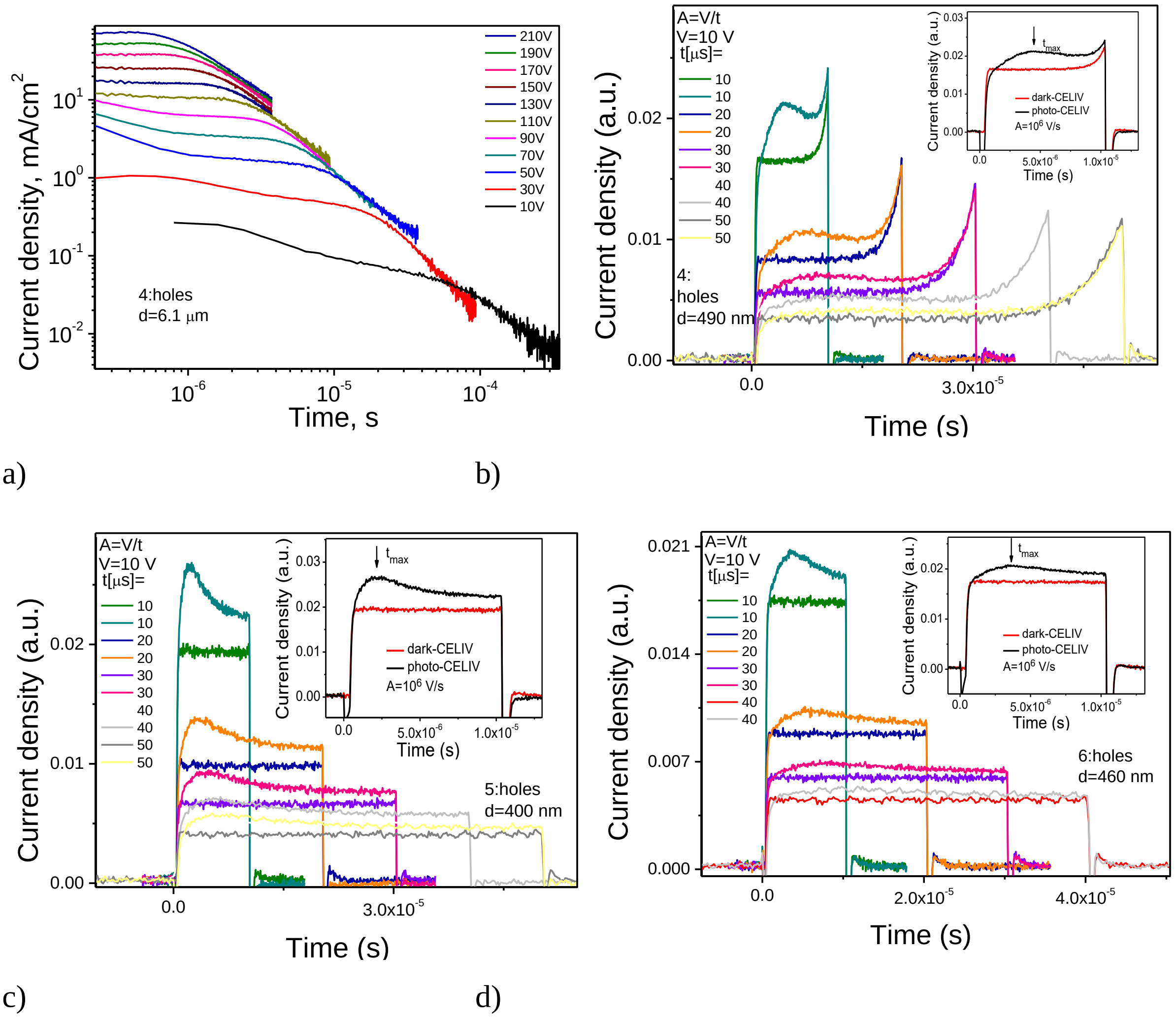

Figure S3: TOF and CELIV current transients for compounds **4**, **5**, and **6**.

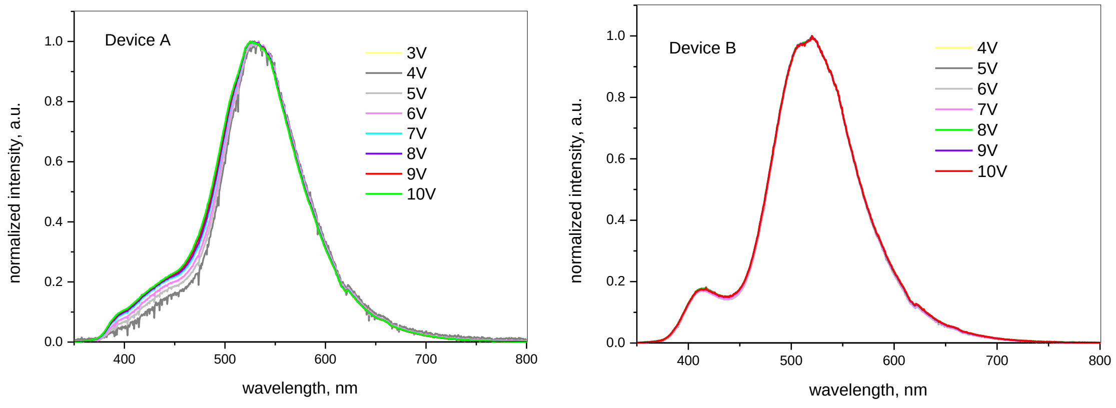

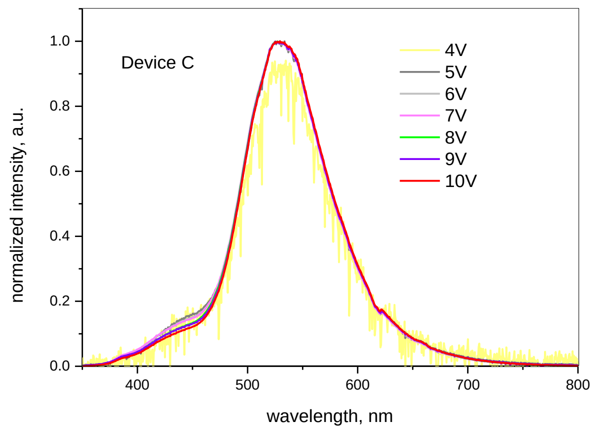

Figure S4: Electroluminescence spectra of the fabricated OLEDs.

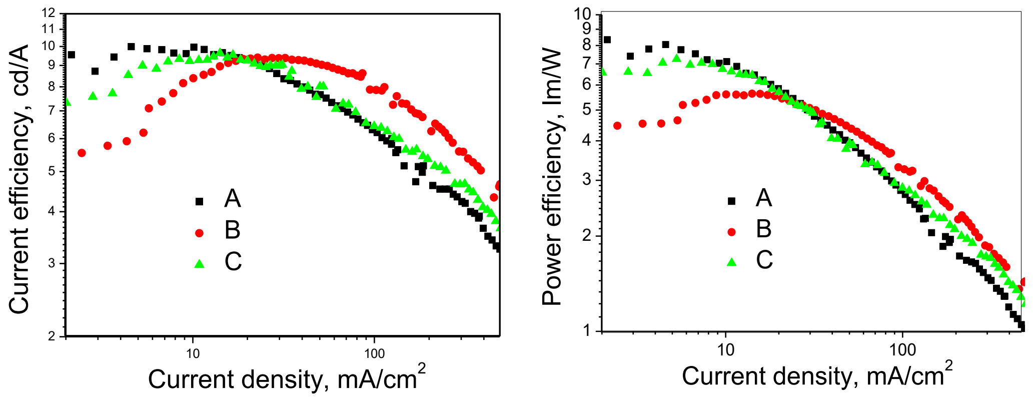

a) b)

Figure S5: Current efficiency (a) and power efficiency (b) versus current density for the tested OLEDs.

References

1. Juška, G.; Genevičius, K.; Viliunas, M.; Arlauskas, K.; Stuchlíková, H.; Fejfar, A.; Kočka, J. *J. Non. Cryst. Solids* **2000**, *266–269 A*, 331–335. doi:10.1016/s0022-3093(99)00720-6
